# Supplementary material for: Evolutionary and cellular analysis of the ‘dark’ pseudokinase PSKH2
Source: Biochem J. 2023 Jan 23;480(2):141–60. doi: 10.1042/BCJ20220474 (PMC9988210; doi:10.1042/BCJ20220474)
Supplement: Supplementary Material [file BCJ-480-141-s1.pdf]

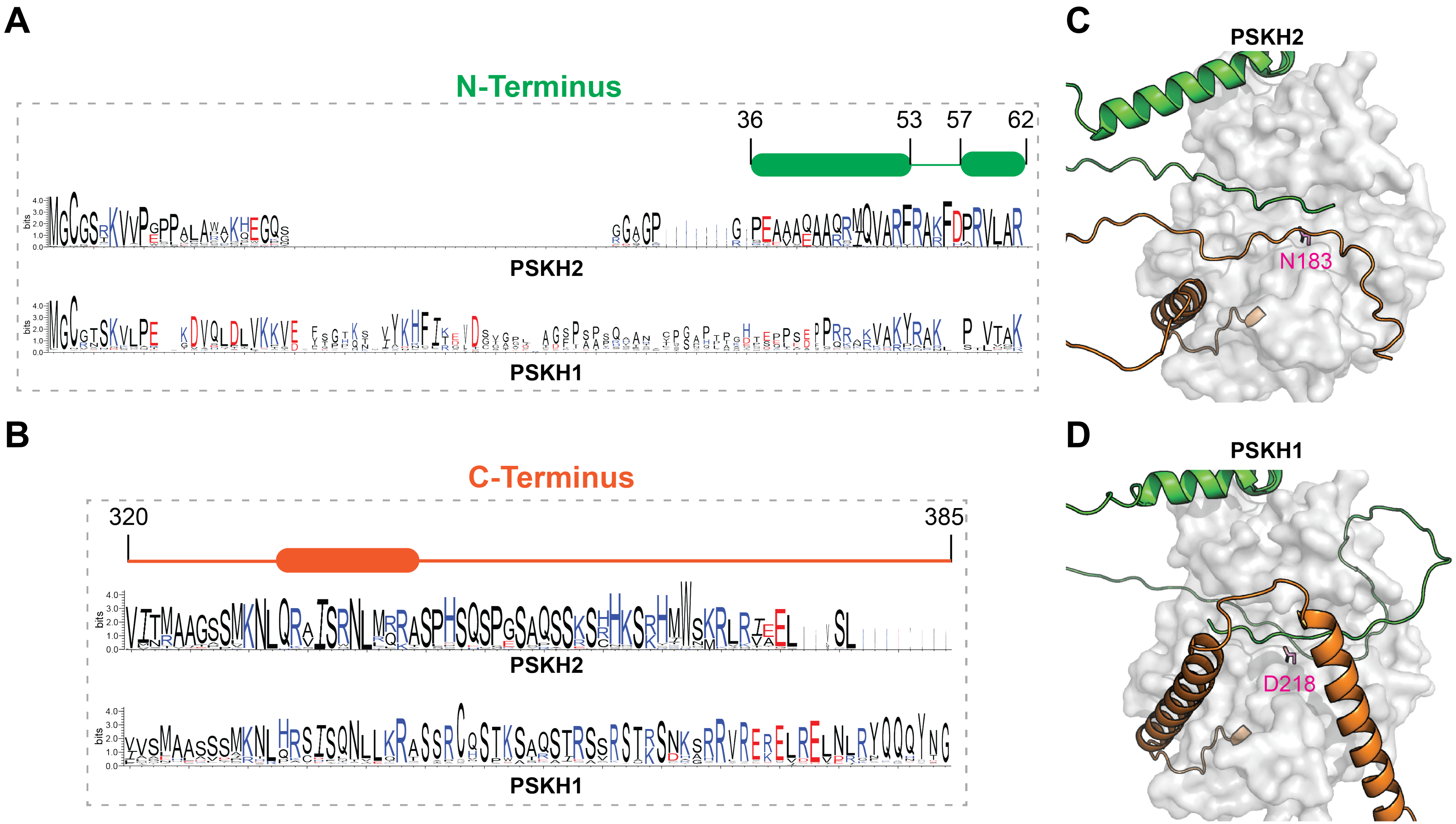

Supplementary Figure 1

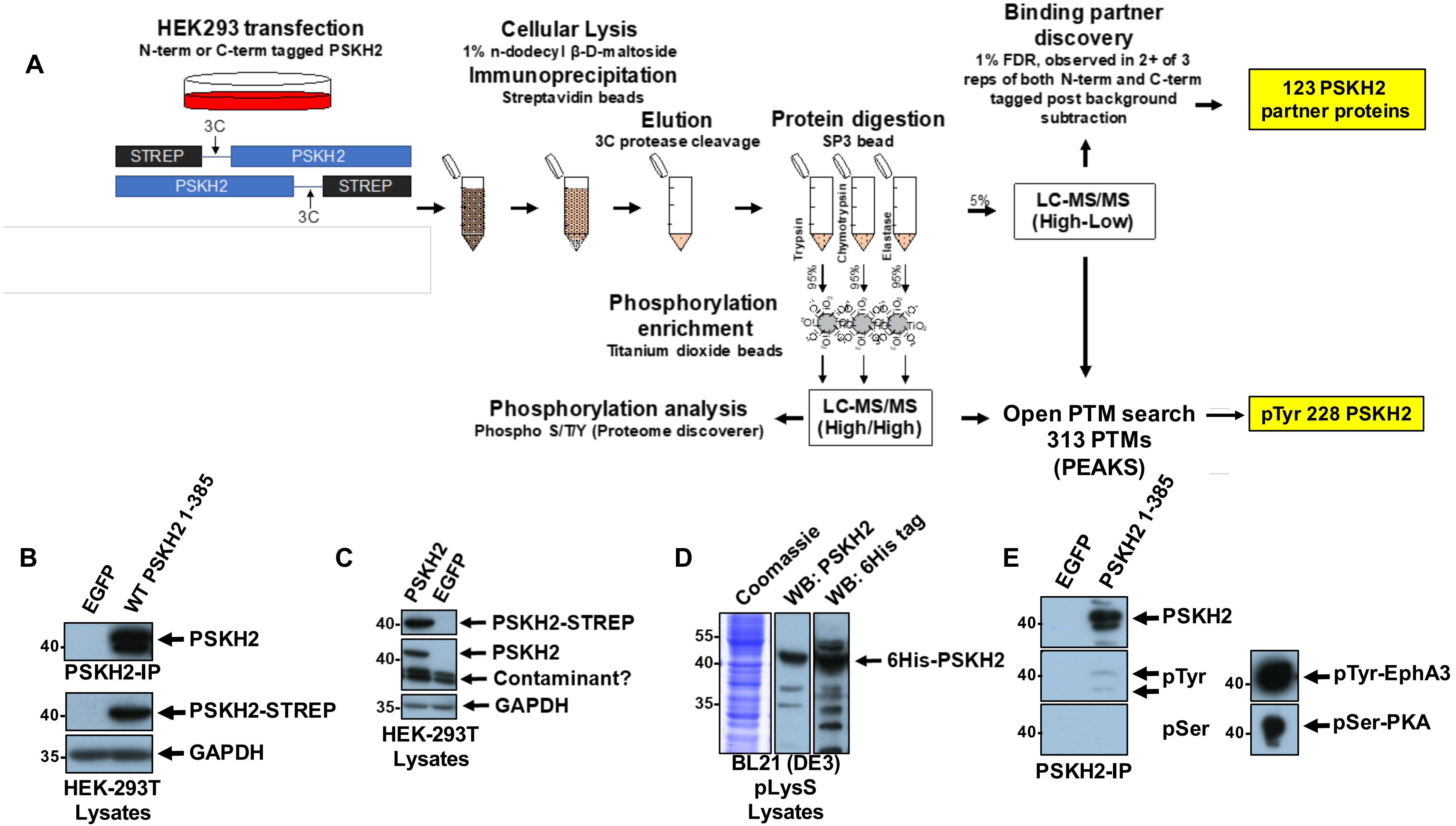

Supplementary Figure 2

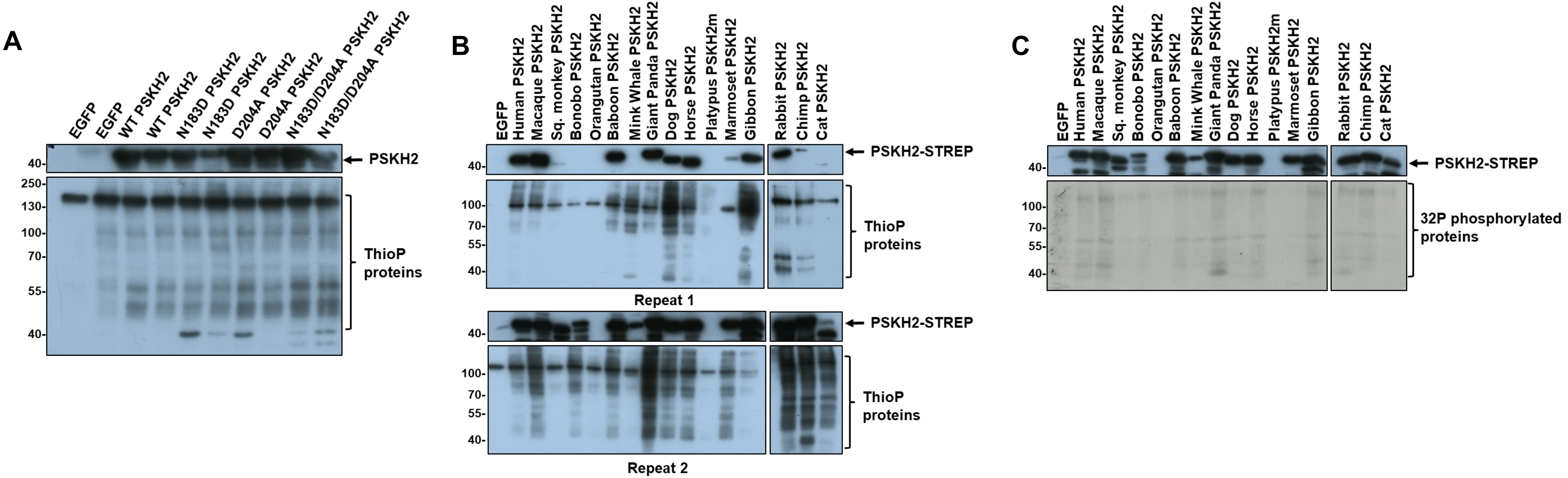

Supplementary Figure 3

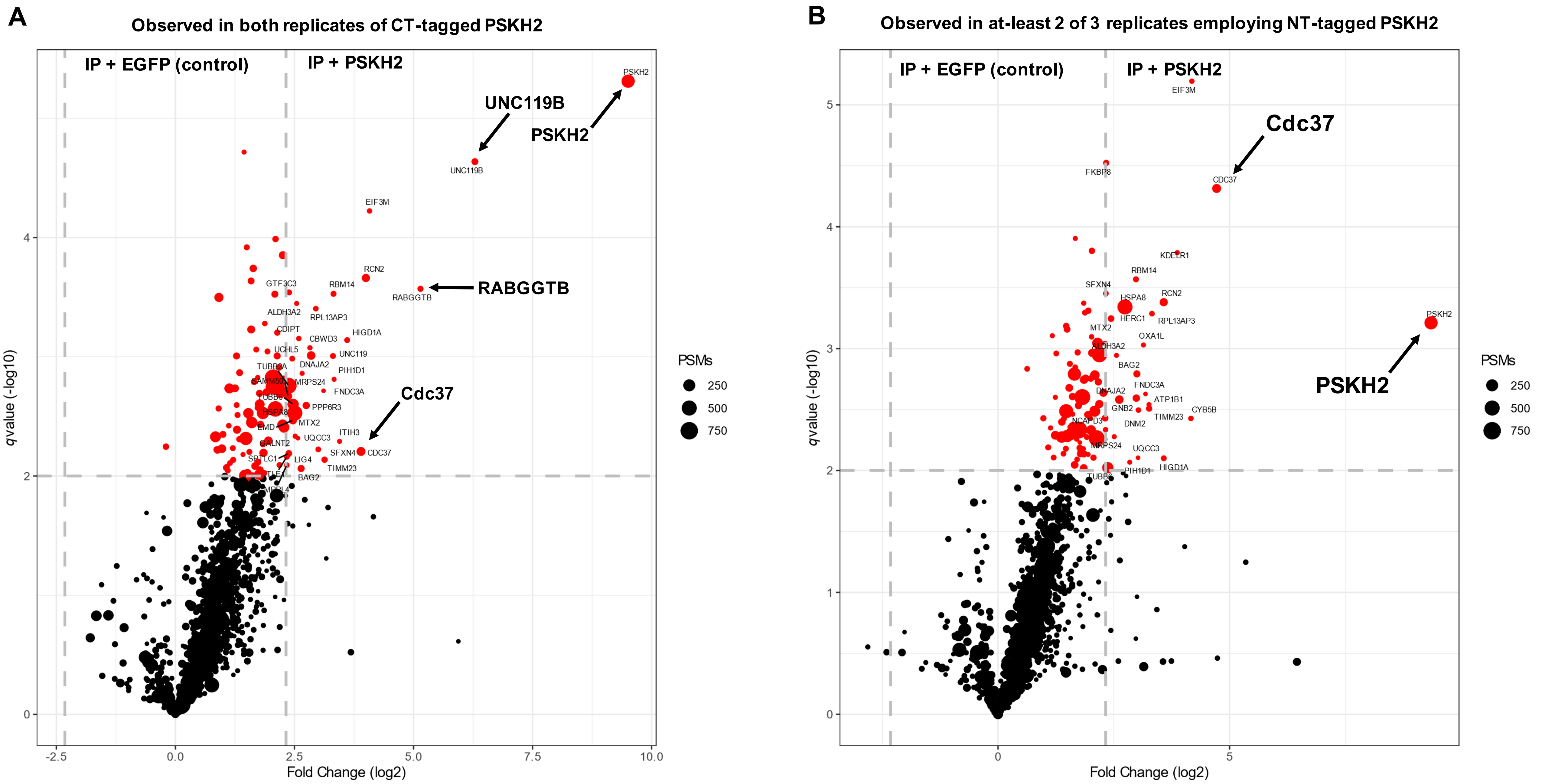

Supplementary Figure 4

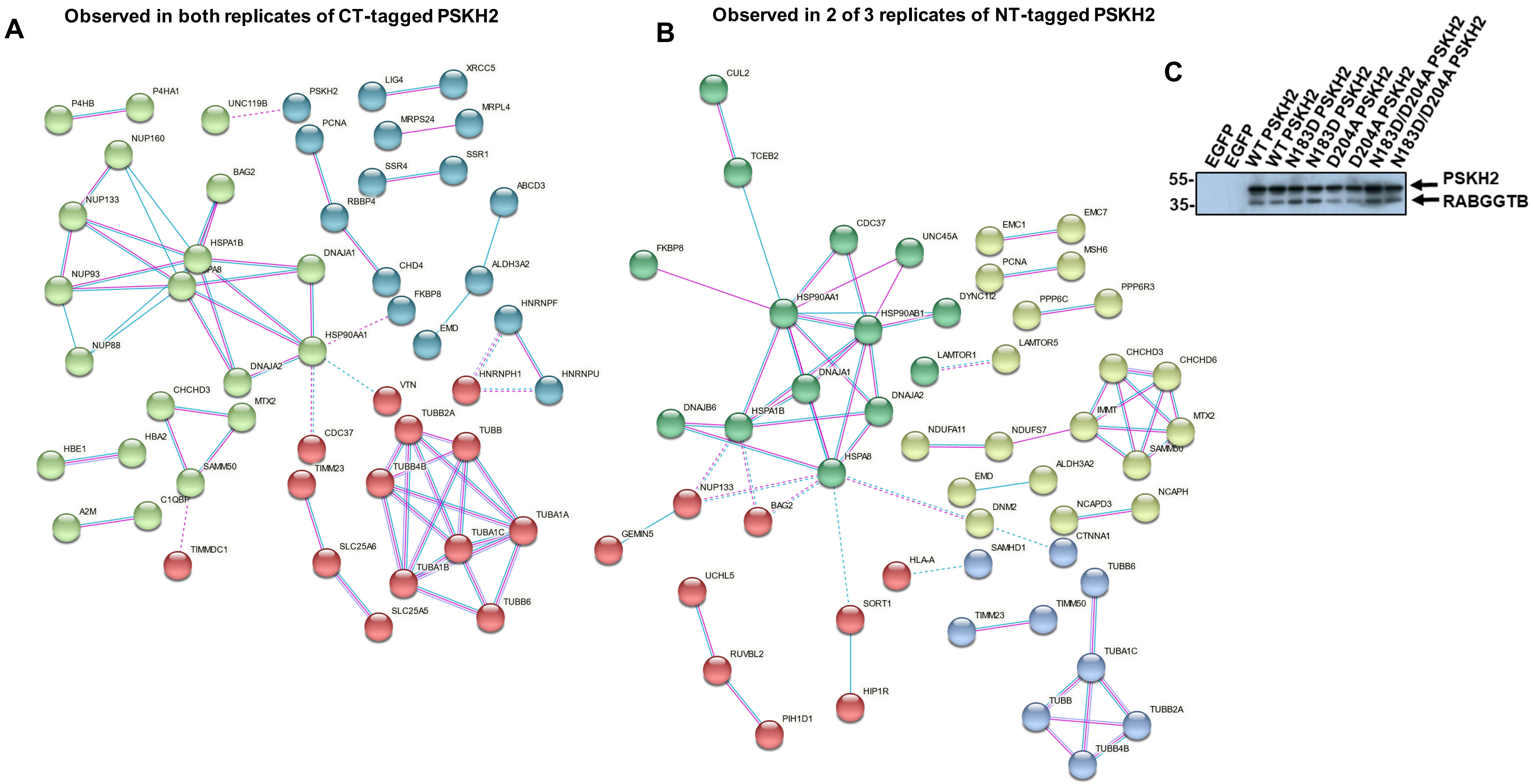

Supplementary Figure 5

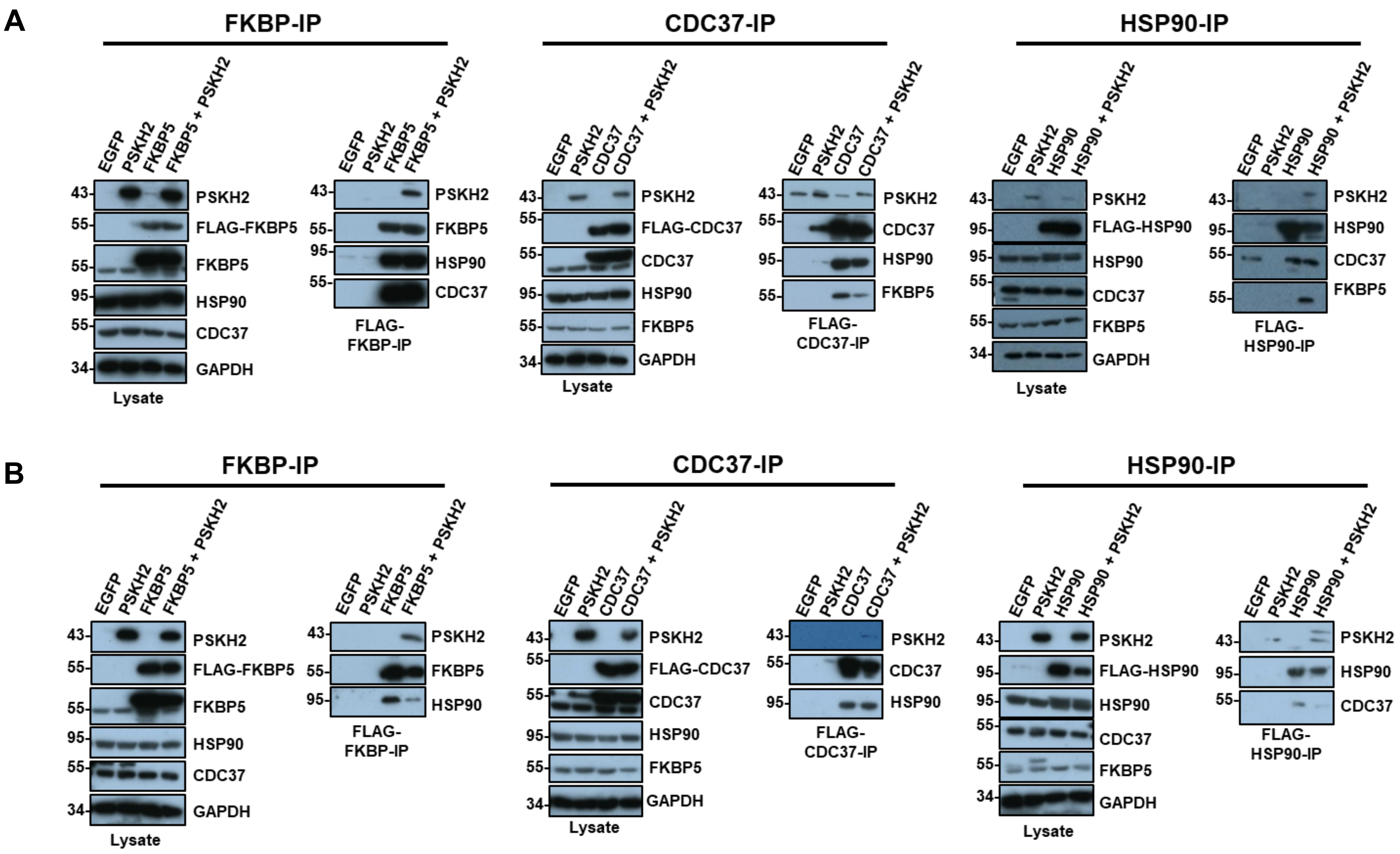

Supplementary Figure 6

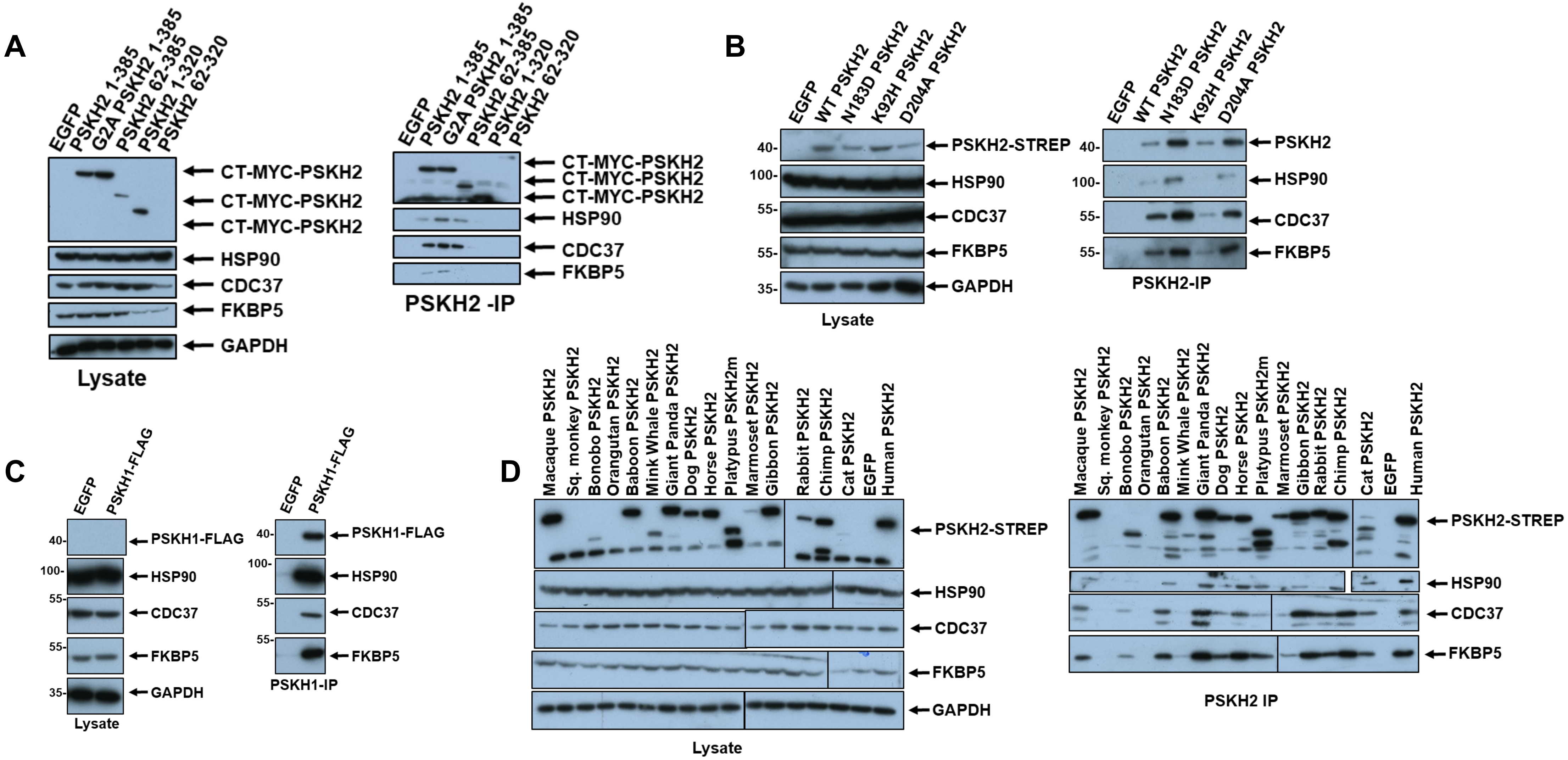

Supplementary Figure 7

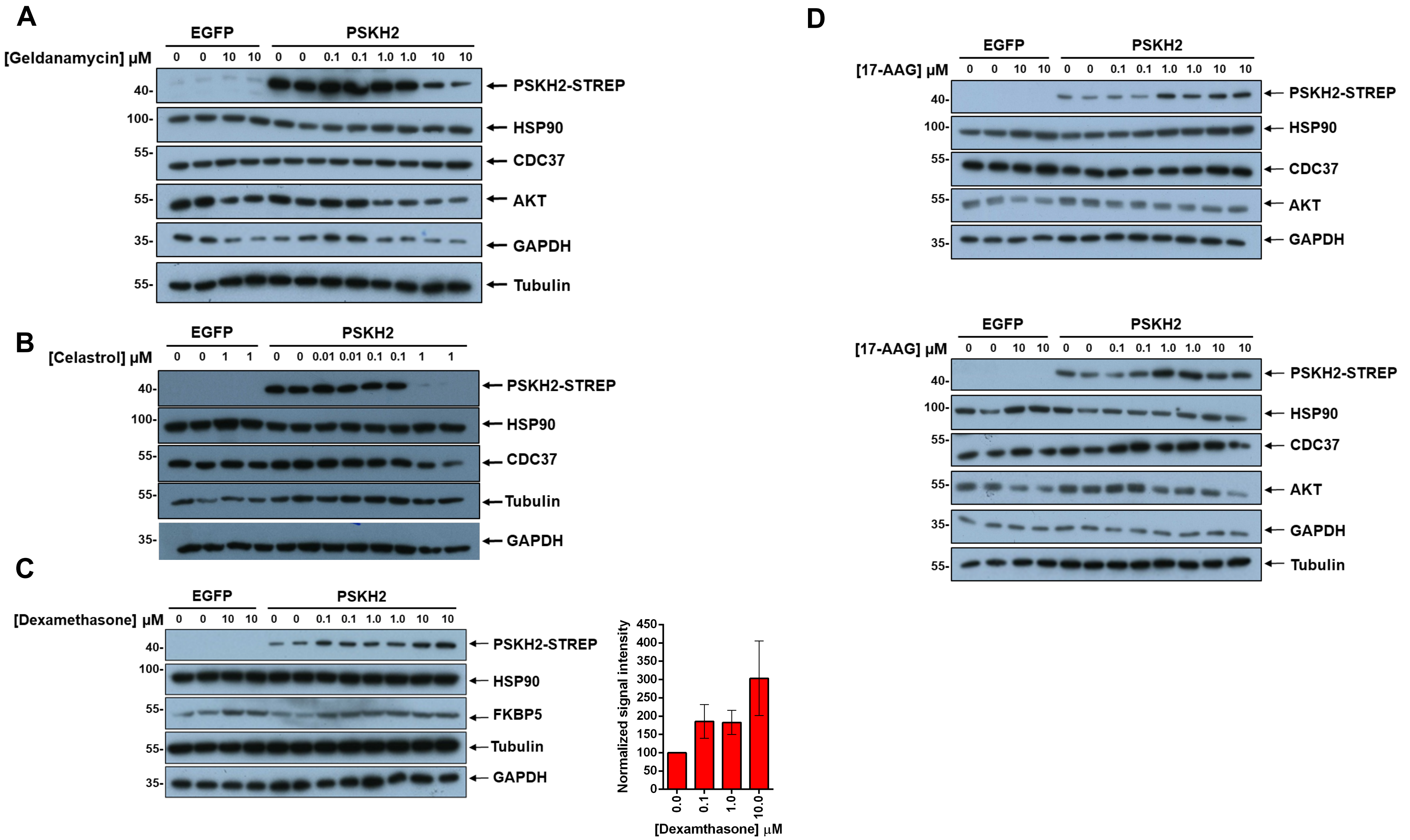

Supplementary Figure 8

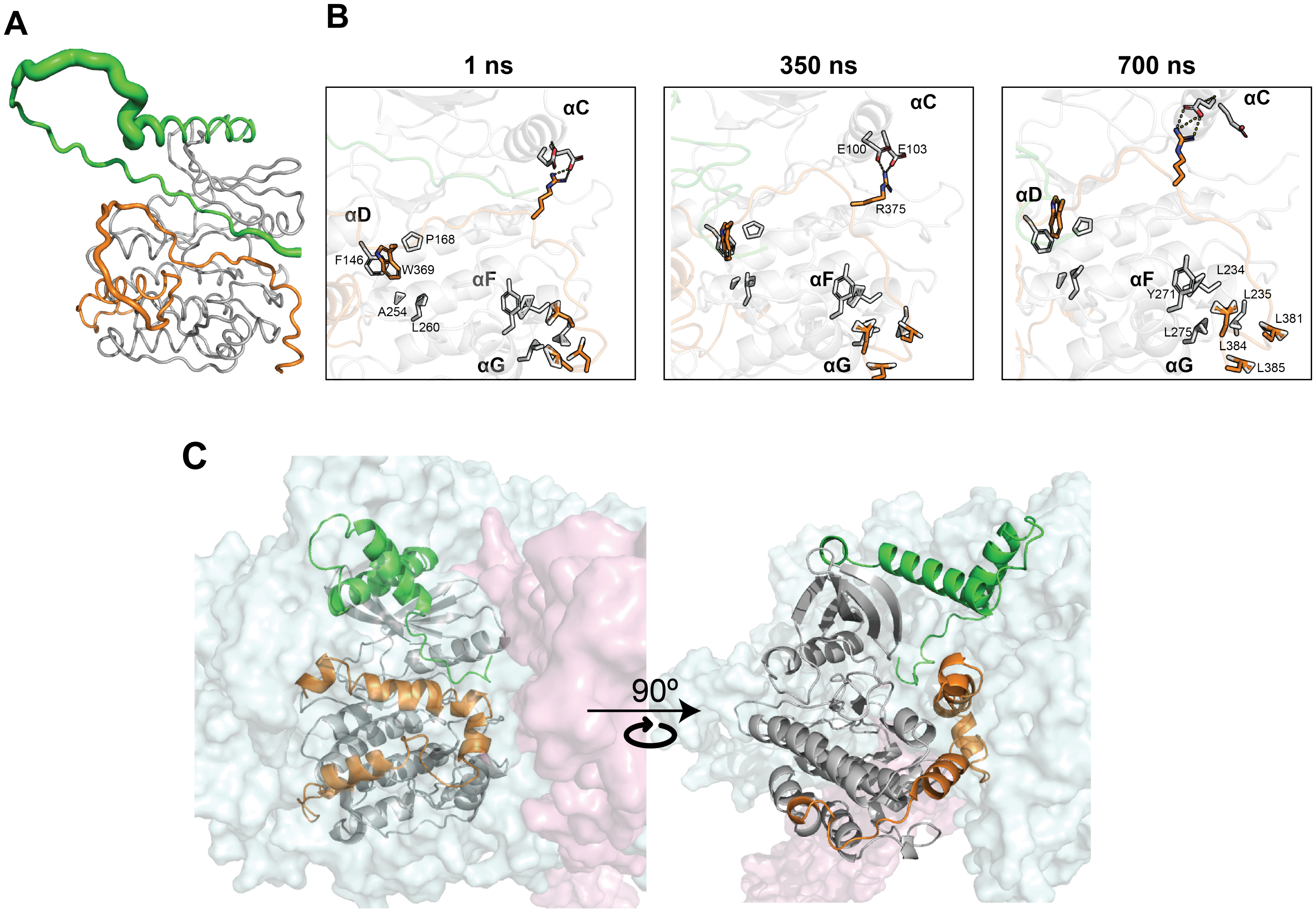

Supplementary Figure 9

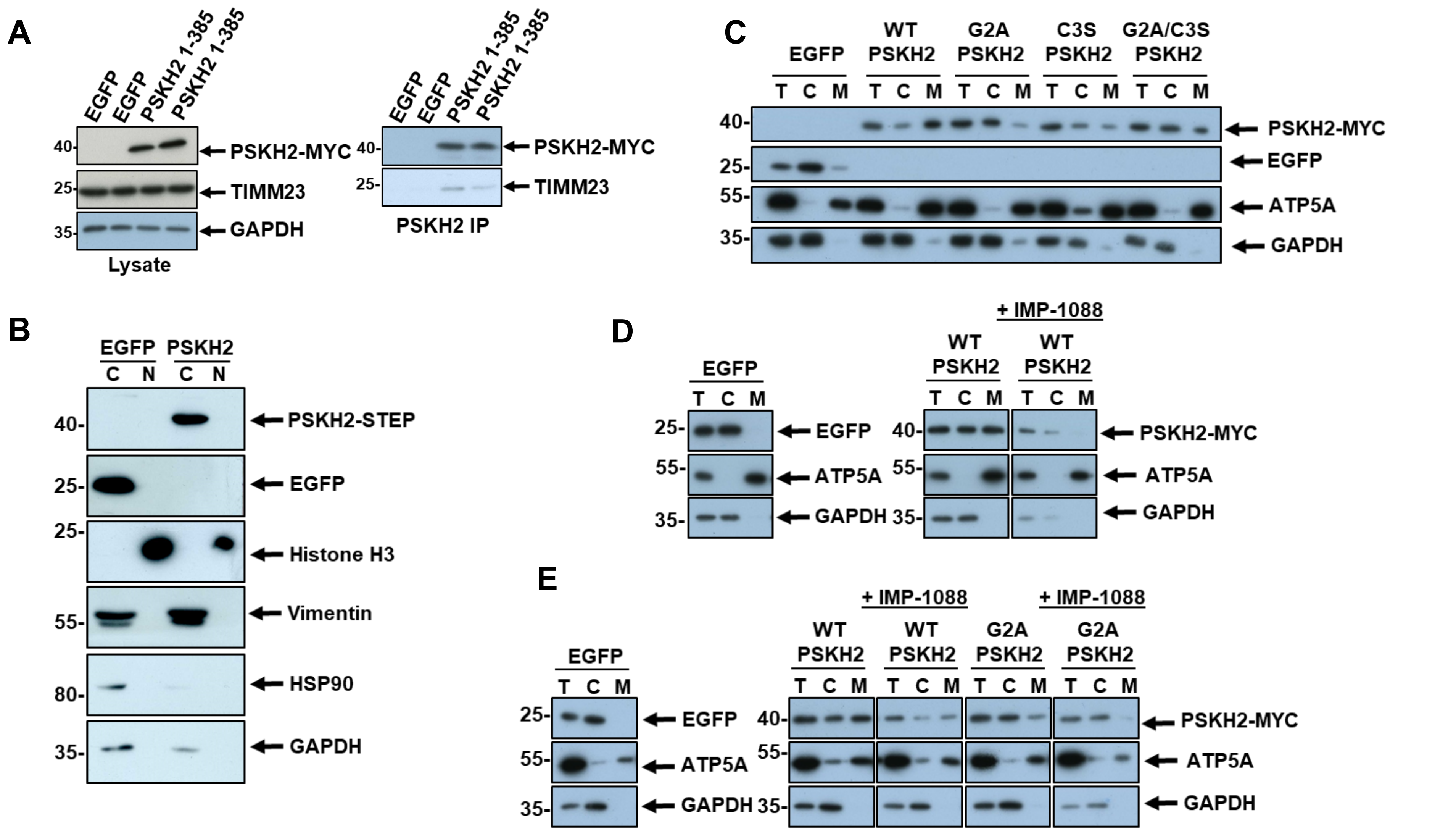

Supplementary Figure 10

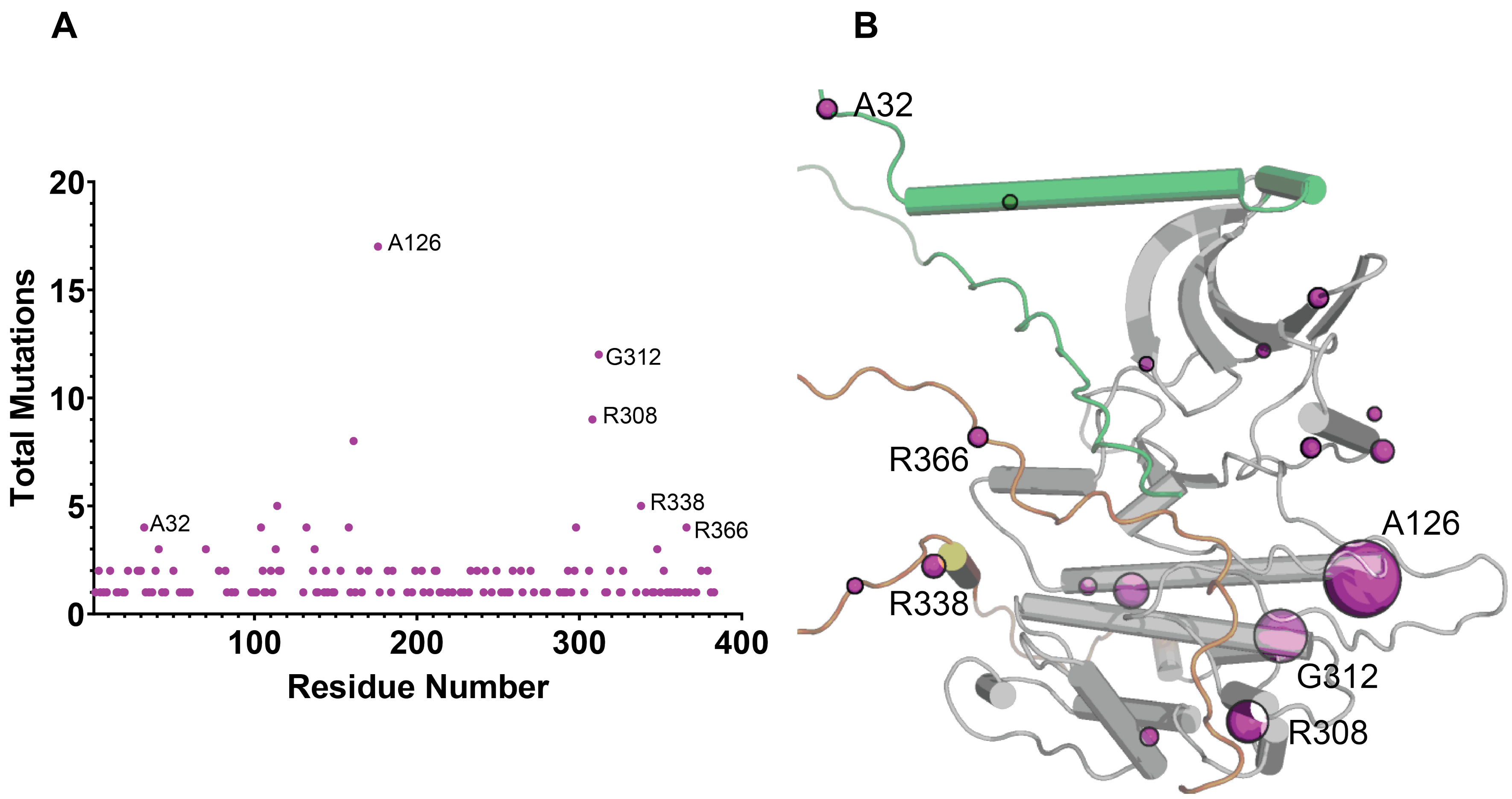

Supplementary Figure 11
